# Editorial Statement and Research Ideas on Using Behavioral Models in Environmental Research and Public Health with Applications

**DOI:** 10.3390/ijerph19127137

**Published:** 2022-06-10

**Authors:** Wing-Keung Wong

**Affiliations:** 1 Department of Finance, Fintech & Blockchain Research Center, and Big Data Research Center, Asia University, Taichung City 41354, Taiwan; wong@asia.edu.tw; 2 Department of Medical Research, China Medical University Hospital, Taichung City 41354, Taiwan; 3 Department of Economics and Finance, The Hang Seng University of Hong Kong, Shatin 999077, Hong Kong

Behavioral models are very important in the development of both environmental research and public health because much of the evidence of empirical findings cannot be explained by using the traditional theories in environmental research and public health; behavioral models play a key role in the analytical apparatus of contemporary approaches to overcome the difficulty in all areas of both environmental research and public health.

To study empirical phenomena in environmental research and public health that cannot be explained by using the traditional theories, scholars can use the ideas of behavioral theory. To do so, scholars can first establish behavioral theories and, thereafter, establish the corresponding statistical estimators and testing statistics in environmental research and public health. They could then conduct simulations to evaluate both power and size of the estimation to find the best estimators or test statistics that are efficient and powerful, and then, obtain estimators that can yield the best forecast values for some future periods. Thereafter, practitioners could apply the models to study some interesting issues in environmental research and public health.

There are many areas for developing new behavioral theories and models in both environmental research and public health. For example, academics can use different utility functions, different heuristics, and different risk measures to develop some sophisticated theories for behavioral models in both environmental research and public health. Scholars could also extend some existing theories, such as stochastic dominance, copulas, wavelet, jump, cointegration, causality, and many others, that fit into the empirical phenomena in environmental research and public health better. 

To develop new behavioral theories and applications and to contribute to the literature in this area, the Special Issue on Behavioral Models in Environmental Research and Public Health with Applications, edited by Wing-Keung Wong, is devoted to the latest advancements in the development of new behavioral models in environmental research and public health with applications in 2021. This Special Issue will also recommend authors to have state-of-the-art practical applications of advanced tools in probability, mathematics, and statistics in environmental research and public health.

We invite academics and practitioners to submit their original articles that fit the spirit and the scope of this Special Issue. We encourage authors to submit papers with sophisticated theories, supported by the latest information and advanced tools, which can be used to obtain better applications of the newly developed behavioral models in both environmental research and public health with applications for any latest issue. 

This Special Issue, “Behavioral Models in Environmental Research and Public Health with Applications”, edited by Wing-Keung Wong, aims at bringing together related theories, practices, and applications that link to the environmental and public health sectors in different countries and is devoted to research for advancements and developments in the environmental and public health sectors by using the advanced tools and the latest behavioral models. This work contributes new developments of behavioral models in environmental research and public health with applications to the literature and provides a useful compendium to serve as a guideline to academics, practitioners, researchers, and policymakers, which serves as the outset to envisage and solicit papers from well-established and well-recognized professionals for their contribution to the newly developed knowledge, with the main focus on quantitative aspects of environmental research and public health with applications. The guest editor has put in great effort in inviting potential authors from different spectrums, including academics, practitioners, and policymakers, to submit articles to fit in the spirit and scope of the issue, screening all submitted articles, inviting well-established reviewers to review the submitted articles, and selecting the best articles among all submitted papers to form the Special Issue. I would like to take this opportunity to thank all generous contributors and reviewers and thank the management team at MDPI. Without their relentless efforts and support, this issue would not be successful. 

This Special Issue is a collection of 12 carefully selected papers for publication, including contributions from Acosta et al. [1], Attiq et al. [2], Attiq et al. [3], Cázares-Manríquez et. al. [4], Du et al. [5], Lv et al. [6], Pentrakan et al. [7], Rjoub et al. [8], Wang et al. [9], Mumtaz et al. [10], Wu et al. [11], and Yao et al. [12]. Among them, Attiq et al. [3] used the partial least squares structural (PLS) model to examine the impacts of financial, cognitive, and emotional concerns of consumers on recycling behavior, reuse, and food waste reduction and among restaurant patrons. They found that anticipated negative emotions of financial concern, awareness of consequences, guilt, and habit influence the behaviors of restaurants’ consumer food waste reduction. On the other hand, Attiq et al. [2] used the PLS structural modeling equation to investigate determinants of the behavior of young consumers on food waste reduction. They found significant impacts from both cognitive and emotional aspects on sustainable food waste reduction behavior.

On the other hand, to examine the relationship between proper return behavior of unused or expired medicines and consequence awareness in the public environment, Lv et al. [6] introduced a chain mediation model that can examine the influence mechanism study of the relationship. The model can also be used to study the moderating effects of personal norms, personal health awareness, and return intention. Acosta et al. [1] employed natural language processing models and the complementary analysis to improve the quality of life of health care professionals in the COVID-19 period and evaluated the satisfaction level of the involved participants to improve future experiences. Wang et al. [9] employed the panel threshold regression model to examine how income inequality affects official corruption and environmental standards.

Moreover, Rjoub et al. [8] employed dynamic, heterogeneous panel-level estimators to investigate both the low and high frequency of the data to examine the relationship between natural resources, government effectiveness, and security threats on the regions’ economic development. Cázares-Manríquez et al. [4] used the risk factors including heart rate, age, respiratory rate, gender, respiratory rate, body mass index, and blood pressure to develop quantitative models to predict cumulative trauma disorders and prevent injuries in manufacturing factory operators. 

In addition, Yao et al. [12] employed spatial econometric analysis to examine the impacts of the characteristics of evolution on the mechanism of Chinese venture capital spatial agglomeration to include the spatial effect. Mumtaz, et al. [10] identify the elements that influence restaurant consumers’ behaviors on food waste reduction, reuse, and recycling; Wu et al. [11] employed the chaos analysis and introduced a game model to predict the choice of travel mode and the impact of low-carbon awareness with simulation; and this manuscript provided an editorial statement and conferred some future research ideas on the use of behavioral models in environmental research and public health with applications.
